# Dual Inhibition of Topoisomerase II and Tyrosine Kinases by the Novel Bis-Fluoroquinolone Chalcone-Like Derivative HMNE3 in Human Pancreatic Cancer Cells

**DOI:** 10.1371/journal.pone.0162821

**Published:** 2016-10-19

**Authors:** Yong-Chao Ma, Zhi-Xin Wang, Shao-Ju Jin, Yan-Xin Zhang, Guo-Qiang Hu, Dong-Tao Cui, Jiang-Shuan Wang, Min Wang, Fu-Qing Wang, Zhi-Jun Zhao

**Affiliations:** 1 Clinical Medical Institute of Luohe Medical College, Luohe, Henan, P.R. China; 2 The First Affiliated Hospital of Luohe Medical College, Luohe, Henan, P.R. China; 3 Tumor Occurrence and Prevention Research Innovation team of Luohe, Luohe, Henan, P.R. China; 4 Basic Medical Institute of ZhengZhou University, ZhengZhou, Henan, P.R. China; 5 Institute of pharmacology of Luohe Medical College, Luohe, Henan, P.R. China; 6 Basic Medical Institute of Luohe Medical College, Luohe, Henan, P.R. China; 7 Institute of Chemistry and Biology of Henan University, Kaifeng, Henan, P.R. China; Duke University School of Medicine, UNITED STATES

## Abstract

Both tyrosine kinase and topoisomerase II (TopII) are important anticancer targets, and their respective inhibitors are widely used in cancer therapy. However, some combinations of anticancer drugs could exhibit mutually antagonistic actions and drug resistance, which further limit their therapeutic efficacy. Here, we report that HMNE3, a novel bis-fluoroquinolone chalcone-like derivative that targets both tyrosine kinase and TopII, induces tumor cell proliferation and growth inhibition. The viabilities of 6 different cancer cell lines treated with a range of HMNE3 doses were detected using the 3-(4,5-dimethylthiazol-2-yl)-2,5-diphenyltetrazolium bromide (MTT) assay. Cellular apoptosis was determined using Hoechst 33258 fluorescence staining and the terminal deoxynucleotidyl transferase (TdT) dUTP nick-end labeling (TUNEL) assay. The expression of activated Caspase-3 was examined by immunocytochemistry. The tyrosine kinase activity was measured with a human receptor tyrosine kinase (RTK) detection kit using a horseradish peroxidase (HRP)-conjugated phosphotyrosine (pY20) antibody as the substrate. The topoisomerase II activity was measured using agarose gel electrophoresis with the DNA plasmid pBR322 as the substrate. The expression levels of the P53, Bax, Bcl-2, Caspase-3, -8, -9, p-cSrc, c-Src and topoisomerase II proteins were detected by western blot analysis. The proliferation of five of the six cancer cell lines was significantly inhibited by HMNE3 at 0.312 to 10 μmol/L in a time- and dose-dependent manner. Treatment of the Capan-1 and Panc-1 cells with 1.6 to 3.2 μM HMNE3 for 48 h significantly increased the percentage of apoptotic cells (P<0.05), and this effect was accompanied by a decrease in tyrosine kinase activity. HMNE3 potentially inhibited tyrosine kinase activity *in vitro* with an IC_50_ value of 0.64±0.34 μmol/L in Capan-1 cells and 3.1±0.86 μmol/L in Panc-1 cells. The activity of c-Src was significantly inhibited by HMNE3 in a dose- and time-dependent manner in different cellular contexts. Compared with the control group, HMNE3 induced increased expression of cellular apoptosis-related proteins. Consistent with cellular apoptosis data, a significant decrease in topoisomerase IIβ activity was noted following treatment with HMNE3 for 24 h. Our data suggest that HMNE3 induced apoptosis in Capan-1 and Panc-1 cells by inhibiting the activity of both tyrosine kinases and topoisomerase II.

## Introduction

**Fig 1:**
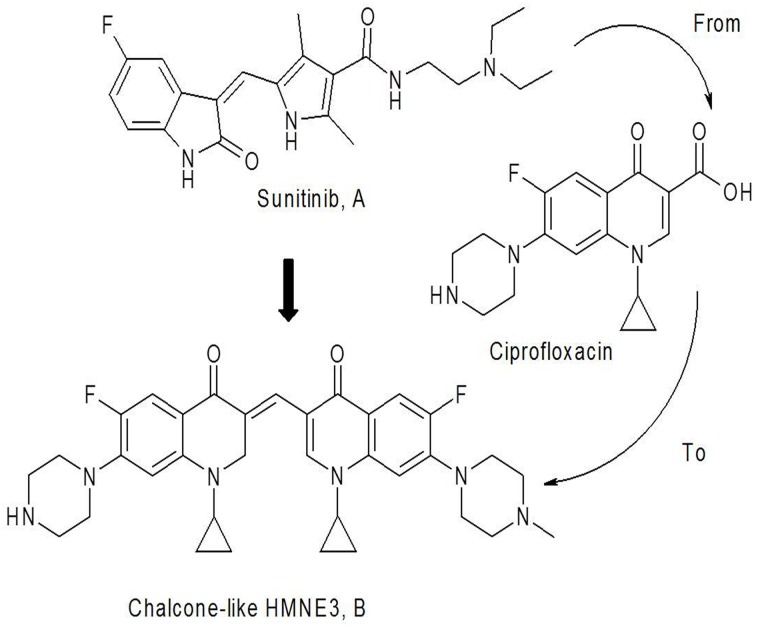
The structure and name of the bis-fluoroquinolone chalcone-like derivative HMNE3. (1-cyclopropyl-3-[1-cyclopropyl-6-fluoro-7-piperazin-1-yl-2,3-dihydro quinolin-4(1H)-one-3-ylidenemethyl]-6-fluoro-7-(4-methylpiperazin-1-yl)-quinolin-4 (1H)-one).

In recent years, multi-target anticancer drugs have become the focus of cancer therapy. Tyrosine phosphorylation plays very important roles in regulating cancer cell behavior, including proliferation, motility and differentiation [1–3]. As receptors for growth factors, including epidermal growth factor (EGF), aberrant signaling of tyrosine kinases has been associated with disease processes, including the development and spread of cancers [4,5]. Sunitinib (Fig 1A) is an oral, multi-target inhibitor of tyrosine kinases that inhibits the activities of c-Src, Bcr-Abl, and other kinases [6, 7]. It has been approved for clinical use in patients with renal carcinoma, as well as neuroendocrine and breast cancers. Its use for treating other solid tumors is currently under investigation. A clinical survey indicated that acquired resistance and toxicities are the main side effects, which limit the use of sunitinib in the treatment of other cancers, particularly pancreatic cancer [8, 9].

Top II has been implicated in multiple cancers due to its involvement in DNA replication, transcription and chromatin remodeling. Specifically, Top IIa has been become a prognostic marker for the prognosis of multiple cancers. Therefore, DNA Top II is a validated target for screening anticancer agents [10, 11]. Top II inhibitors are more efficient in chemotherapy and the most effective among these agents. In the clinic, Top II inhibitors, such as etoposide, have been used to treat human cancers [12]. However, similar to other anticancer drugs, most Top II inhibitors also produce severe side effects, including cardiotoxicity and multidrug resistance. Hence, there is an urgent need for novel Top II-targeting drugs with low toxicity and fewer side effects. Recent studies have demonstrated that antibacterial fluoroquinolones have a potential role in inhibiting tumor cell proliferation, based on the mechanistic similarities and sequence homologies to the drugs targeting eukaryotic topoisomerases [13].

Chemically, sunitinib is an α, β-unsaturated ketone (chalcone) derived from an aldol condensation reaction of fluoro-oxindole with the amide pyrrole aldehyde. Based on the principles of bioisosterism and pharmacophore hybrids in rational drug design, a unique design attempted to replace the oxindole and pyrrole scaffolds with the respective fluoroquinolone and fluoroquinolone aldehyde to create a novel fluoroquinolone chalcone-like derivative. Therefore, we designed and synthesized a series of α, β-unsaturated ketone derivatives, including HMNE3, which retain the structural characteristics of sunitinib, the basic structure of the α, β-unsaturated ketone of tyrosine kinase inhibitors, and the typical fluoroquinolone structure of topoisomerase inhibitors (Fig 1B). These compounds displayed potent cytotoxicity against the tested cancer cell lines *in vitro*. In the present study, we detected the mechanisms of HMNE3-mediated inhibition of cancer cell proliferation using human pancreatic cancer cells. These findings suggest a molecular mechanism of action of HMNE3 that has important implications for pancreatic tumor treatment. This chemical is the first reported dual tyrosine kinase and topoisomerase II inhibitor. Thus, HMNE3 is a promising new therapeutic agent for treating human pancreatic cancer.

## Materials and Methods

### Chemicals

The chalcone-like derivative HMNE3 was designed and synthesized by the Institute of Chemistry and Biology of Henan University. The purity was greater than >98%, as determined by high-performance liquid chromatography (HPLC) analysis. The compound was dissolved in dimethyl sulfoxide (DMSO, Solarbio Science & Technology Co., Ltd.), and the stock solution was 10 mM. The final concentration of DMSO was not greater than 0.1% in the medium, which did not affect cell viability. The HMNE3 structure is illustrated in Fig 1.

### Cell culture

Six human cancer cell lines, T24, Panc-1, Capan-1, DU145, HGC-27, and BGC-823, were originally purchased from the American Type Culture Collection and cultured in DMEM or RPMI1640 medium supplemented with 10% fetal bovine serum and 1% penicillin/streptomycin solutions (Gibco, USA) at 37°C in a 5% CO_2_ and 95% humidified atmosphere and harvested with trypsin-EDTA. Logarithmically growing cells were used for all experiments. The cells were cultivated in 75-cm^2^ flasks (Costar, Cambridge, MA) until they reached approximately 50 to 70% confluence. The cells were then treated with varying doses of the indicated chemicals. DMSO alone was used as the vehicle control.

### MTT assay

The cells were seeded at a density of 1×10^3^ cells/mL per well in 96-well culture plates for 24 h. Then, the cells were treated with the indicated amounts of HMNE3. The wells containing cells that were incubated with medium only were used as the control group. At 24, 48 and 72 h, 20 μL of MTT (5 mg/mL, 3-[4,5–dimethylthiazol-2-yl]-2,5-diphenyltetrazolium bromide; Sigma, St. Louis, MO, USA) was added to the cells and incubated for 4 h at 37°C. Then, the supernatant was removed, and 150 μL of DMSO was added. The plates were rocked until the blue crystals were dissolved in DMSO. The optical density (OD) was then detected at a wavelength of 570 nm using a 96-well multiscanner autoreader (Bio-Rad, USA). The following formula was used: inhibition of cell proliferation (%) = [1-(OD of the experimental samples/OD of the control)]×100. The IC_50_ values were defined as the concentration at which cell proliferation was inhibited by 50% and were calculated using sigmoidal inhibitory response curves and GraphPad Prism software. The data are presented as the means ± standard deviation (SD) (n = 3). All proliferation assays were performed in triplicate, and each sample was repeated thrice.

### Cell cycle analysis

The cells were seeded on 6-well plates (Corning Incorporated), cultured and treated as previously described. Following the treatments, both the adherent and non-adherent cells were collected, washed once in cold 1× phosphate-buffered saline (PBS) and fixed in 50% ethanol overnight at 4°C. The cells were stained with a propidium iodide solution containing RNase A (Sigma) for 15 min at 37°C. The DNA content was analyzed using a FACS Caliber cytometer.

### Hoechst 33258 staining

The Capan-1 and Panc-1 cells (5×l0^3^ cells/mL) were seeded on 35-mm glass slides that were preincubated with 0.01% poly-L-lysine. After a 48-h treatment with HMNE3, the cells were washed twice with PBS and incubated with 5 μg/mL Hoechst 33258 (Sigma, St. Louis, MO, USA) for 10 min at 37°C in the dark. The cells were then washed and fixed with 4% paraformaldehyde in PBS for 5 min at 4°C. The nuclear morphology was then examined under a fluorescent microscope (BX51, Olympus, Japan).

### Analysis of apoptosis using the TUNEL assay

Logarithmically grown cells were treated as previously described. After a 48-h treatment with the indicated doses of HMNE3, the apoptotic cells were examined using the terminal deoxynucleotidyl transferase-mediated dUTP nick-end labeling (TUNEL) assay (Promega, Madison, WI, USA) according to the manufacturer’s instructions. The cells with apoptotic DNA strand breaks were analyzed by detecting their fluorescence. To examine the total cell numbers, the nuclei were simultaneously labeled with 4’, 6-diamidino-2-phenylindole (DAPI). Merged images of both channels were captured at 100× magnification using a fluorescence microscope (BX51, Olympus, Tokyo, Japan). The percentage of apoptotic cells was determined by counting the number of apoptotic cells and dividing this value by the total number of cells in the areas. At least 10 randomly chosen areas were evaluated per slide. Each observer was blinded to the other cellular data as well as to the results of the other observer.

### Immunocytochemical assay

For the immunocytochemical analysis, 2×10^4^ cells were plated on coverslips placed in 12-well plates. All of the cells were treated as previously described. The cells were fixed with 4% paraformaldehyde in PBS for 15 min. The fixed cells were then washed with PBS and incubated with 5% bovine serum albumin (BSA) diluted in PBS for 30 min. The cells were incubated with a rabbit anti-human cleaved Caspase-3 antibody (Asp175; Cell Signaling Technology, Inc.; diluted 1:100 in 0.5% BSA/PBS) overnight at 4°C, washed with PBS, and incubated with a biotinylated horse anti-rabbit IgG antibody (1:200; Vector Laboratories, USA) in 0.5% BSA/PBS for 30 min. The negative controls were performed by incubating the cells with a non-immune rabbit IgG. After rinsing with PBS for 5 min, the streptavidin-horseradish peroxidase enzyme complex (1:250; Vector Laboratories, USA) was diluted in PBS and incubated with the cells for 30 min. After washing in PBS (3×5 min), staining was visualized by incubation of the cells with 3, 3- diaminobenzidine (DAB) and counter-staining with a hematoxylin solution (Sigma-H9627). The slides were washed with PBS (3×5 min) and mounted. The cells were imaged using a microscope (Nikon TE300). Image-Pro Plus software version 6.0 was used to quantify the number of cells, the areas stained, and the staining intensity.

### ELISA kinase assay

Enzyme-linked immunosorbent assays (ELISAs) were used to detect the effects of HMNE3 on the activities of various tyrosine kinases. Ninety-six-well plates were pre-coated with 20 μg/mL of a 4:1 poly (Glu,Tyr) solution (Sigma, St. Louis, MO, USA) as a substrate. An ATP solution (10 μmol/L) was added to each well. Varying doses of HMNE3 were added to each test well. The kinase reaction was initiated by the addition of purified tyrosine kinase proteins diluted in 49 μL of kinase reaction buffer. After incubation for 1 h at 37°C, the plate was washed thrice with PBS containing 0.1% Tween-20 (PBST). An anti-phosphotyrosine (PY99) antibody [100 μL; 1:500, diluted in 5 mg/mL BSA-PBST] was then added. After 30 min incubation at 37°C, the plate was washed thrice, and 100 μL of a horseradish peroxidase-conjugated goat anti-mouse IgG (1:5,000, diluted in 5 mg/mL BSA) was added. The plate was then incubated at 37°C for 30 min and washed thrice. The reaction was terminated by the addition of 50 μL of 2 mol/L H_2_SO_4_, and the plate was read using a multi-well spectrophotometer (Bio-Tek, USA) at 490 nm. The inhibition rate (%) was calculated using the following equation: [1-(A490/A490 control)]×100%. The IC_50_ values were calculated from the inhibition curves in two separate experiments.

### Supercoiled pBR322 DNA relaxation assay

DNA topoisomerase II activity was determined using the supercoiled pBR322 DNA relaxation assay as previously described [14]. Briefly, 0.25 μg of pBR322 DNA was incubated with 1 unit of human Top IIα (Sigma, St. Louis, MO, USA) and 1.6 μM HMNE3 for 30 min at 37℃ in a total of 20 μL of reaction buffer [10 mM Tris (pH 7.9), 50 mM KCl, 50 mM NaCl, 5 mM MgCl_2_, 0.1 mM EDTA, 15 μg/mL BSA, and 1 mM ATP]. The reaction was then stopped with 2 μL of 10% sodium dodecyl sulfate (SDS) and 1 μL proteinase K (1×10^4^ mg/L). The samples were subjected to electrophoresis on a 0.8% agarose gel in 1× TAE at 5 V/cm for 1 h. Then, the gel was stained with 0.5 μg/mL of ethidium bromide (EB) for 30 min, destained with distilled water for 30 min, and photographed under a UV trans-illuminator.

### kDNA Decatenation Assay

The ATP-dependent decatenation of kinetoplast DNA (kDNA) was used for detection the Topo II activity as the references described [15]. Firstly, untreated and treated cells were harvested on ice. Then, nuclear extracts were separated from whole cellular proteins. Total 0.1 μg kDNA and 1unit of human topo IIa (TopoGEN, Inc., Columbus, OH) were mixed with the reaction buffer consisting of 50 mmol/L Tris-HCl (pH 7.7), 120 mmol/L KCl, 10 mmol/L MgCl_2_, 1mmol/L ATP, 0.5 mmol/L DTT, 0.5 mmol/L EDTA, and 30 μg/mL bovine serum albumin. After incubation at 37°C for 15 min, the reaction was stopped by the addition of 10% SDS (1 μL). The DNA samples were subjected to electrophoresis in a 1% agarose gel in 1×TAE at 4 V/cm for 2 h.

### Western blot

The cells were washed twice with ice-cold PBS and then lysed with RIPA buffer [20 mmol/L Tris-HCl (pH 7.5), 150 mmol/L NaCl, 1 mmol/L EDTA (disodium salt), 1 mmol/L EGTA, 1% Triton X-100, 2.5 mmol/L sodium pyrophosphate, 1 mmol/L β-glycerophosphate, 1 mmol/L Na_3_VO_4_, 1 μg/mL leupeptin, and 1 mmol/L phenylmethylsulfonyl fluoride]. The protein concentrations in the cell lysates were determined with a BCA protein assay kit (Thermo Fisher Scientific), and equal amounts of proteins were subjected to sodium dodecyl sulfate polyacrylamide gel electrophoresis (SDS-PAGE) on 8 or 12% polyacrylamide gels. The separated proteins were electrophoretically transferred to polyvinylidene fluoride membranes (Millipore, USA). The membranes were then incubated with blocking solution (5% skim milk in PBST) for 1 h at room temperature before incubation with the primary antibodies overnight at 4°C. The antibodies against phosphorylated c-Src (Y416), total c-Src, and topoisomerase II were obtained from Cell Signaling Technology. The antibodies against P53, Bax, Bcl-2, and Caspase-3, -8, and -9 were purchased from Santa Cruz Biotechnology. The anti-β-actin antibody was purchased from Sigma. After three washes (3×5 min) with PBST, the membrane was incubated with horseradish peroxidase–conjugated antibodies against rabbit or mouse IgG (GE Healthcare) for 1 h at room temperature. The immune complexes were then detected with enhanced chemiluminescence (Thermo Scientific, Rockford, USA) using an LAS-3000 (Fuji Film, Japan) and analyzed with ImageJ software to measure the band intensity and determine the relative protein abundance.

### Statistical analysis

All data are presented as the means ± SD (n = 3 to 6). Student’s two-tailed t test was performed to determine the significant differences between the treatment and control groups. Significance was set at the P<0.05 level (symbols indicate data that are statistically significant).

## Results

### The characterization of HMNE3

In our present study, we have designed and synthesized twenty α, β-unsaturated ketone derivatives, which retain the structural characteristics of sunitinib (Fig 1A), and the typical fluoroquinolone structure of topoisomerase inhibitors (Fig 1B). In our preliminary MTT assay, we found these compounds displayed potent cytotoxicity against the tested cancer cell lines *in vitro*. However, among the 20 derivatives, HMNE3 showed a stronger cytotoxicity against cancer cells. So, HMNE3 was selected as the candidate for exploring the mechanisms of anti-tumor.

### The proliferation of six different cell lines was significantly inhibited by HMNE3

**Fig 2:**
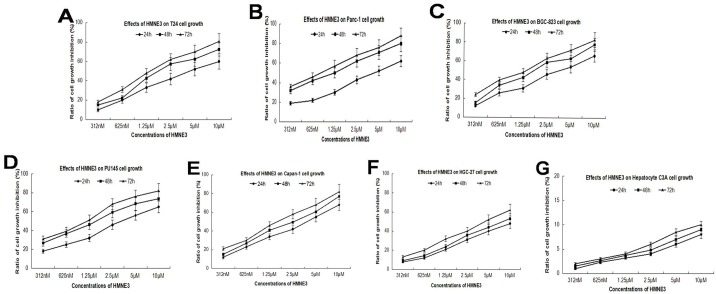
Effects of HMNE3 on cancer cell proliferation. The logarithmically growing cell lines (T24, Panc-1, BGC-823, PU145, Capan-1, HGC-27, and normal hepatocyte C3A) were treated with various concentrations of HMNE3 for 24, 48 and 72 h. The inhibition of cell proliferation was assessed with the MTT assay, and the data were presented as the means of at least three independent experiments.

To detect the effect of HMNE3 on proliferation of cancer cells, we selected six different cell lines and treated them with varying concentrations of HMNE3. As shown in Fig 2A–2F, five of the six cell lines were significantly inhibited by HMNE3 in a time- and dose-dependent manner. However, no effect was noted in the C3A normal human hepatocyte line (Fig 2G). The IC_50_ values are presented in Table 1.

**Table 1:** The IC_50_ values of HMNE3 in six cell lines following treatment for 48 h.

Cell lines	Panc-1	T24	BGC-823	PU145	HGC-27	Capan-1
**IC50**	3.15 μM	3.65 μM	3.8 μM	3.2 μM	5.6 μM	2.9 μM

### HMNE3 treatment induced cell cycle arrest in Capan-1 cells

**Fig 3:**
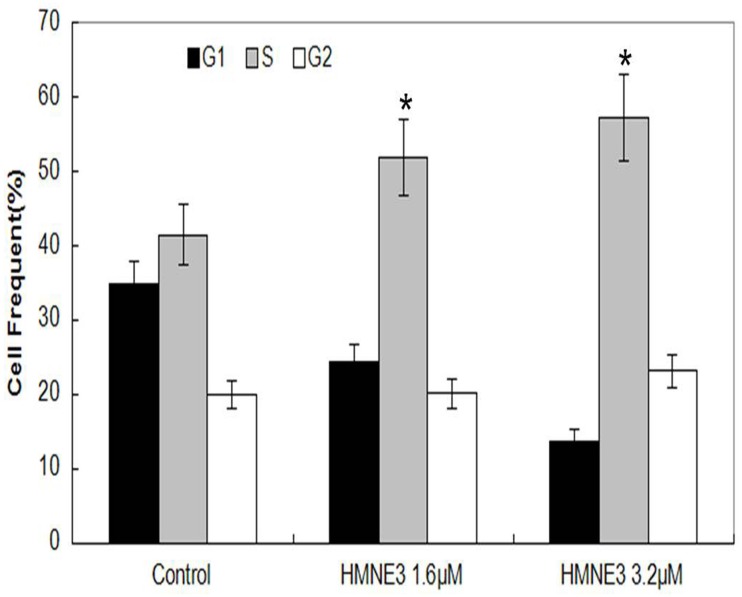
Role of HMNE3 in the cell cycle progression of Capan-1 cells. Logarithmically growing Capan-1 cells were treated with 1.6 μM or 3.2 μM HMNE3 for 24 h. Cell cycle was analyzed by flow cytometry. The statistics of three independent experiments are presented. *, P<0.05 compared with the control group; Student’s t test.

Both tyrosine kinase and Top II inhibitors can cause typical cell cycle arrest. However, the effects of dual tyrosine kinase / Top II inhibitors on cell cycle progression remain unknown. Our results indicate that HMNE3 caused a typical S phase arrest in Capan-1 cells in a dose-dependent manner (Fig 3).

### HMNE3 treatment induced Capan-1 cell apoptosis

**Fig 4:**
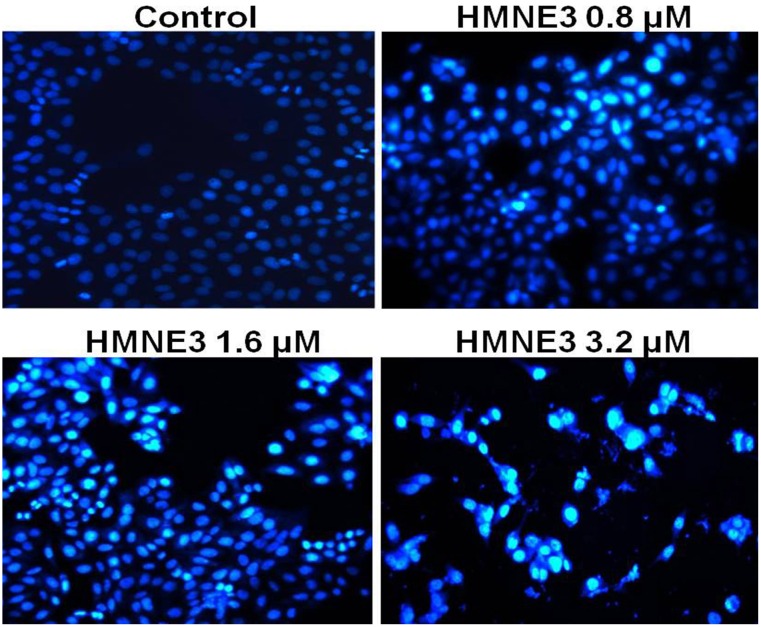
Nuclear staining of Capan-1 cells following 48 h treatment with HMNE3. Approximately 5×10^3^ cells/mL were seeded on 35-mm glass slides. After treatment with HMNE3 for 48 h, the cells were washed twice with PBS and incubated with 5 μg/mL Hoechst 33258 for 10 min at 37°C in the dark. The nuclear morphology was then examined under a fluorescent microscope.

**Fig 5:**
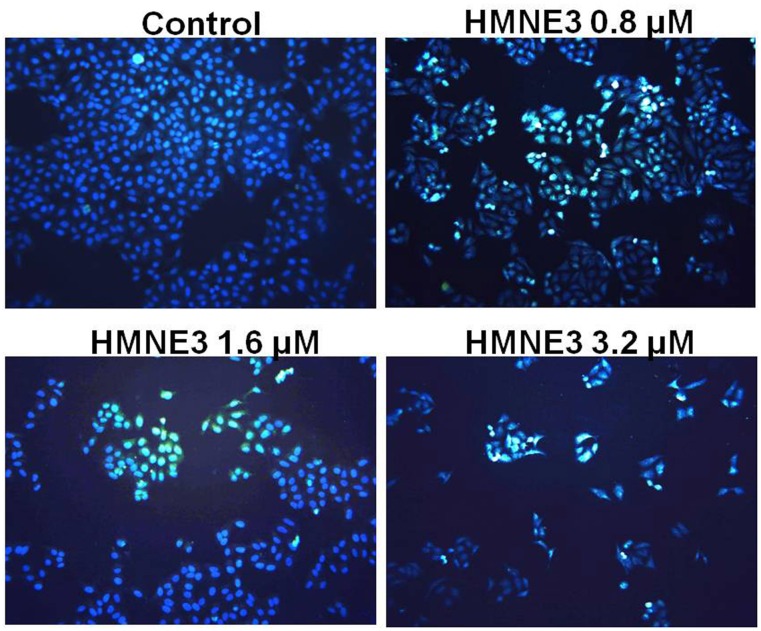
TUNEL staining of Capan-1 cells following a 48 h treatment with HMNE3. Logarithmically growing cells were treated with the indicated doses of HMNE3 for 48 h, and the apoptotic cells were examined using the TUNEL assay. Cells from at least five randomly chosen areas on each slide were visualized and photographed using a fluorescent microscope. The percentage of apoptotic cells was determined by counting the number of apoptotic cells and dividing this value by the total number of cells in the areas.

**Fig 6:**
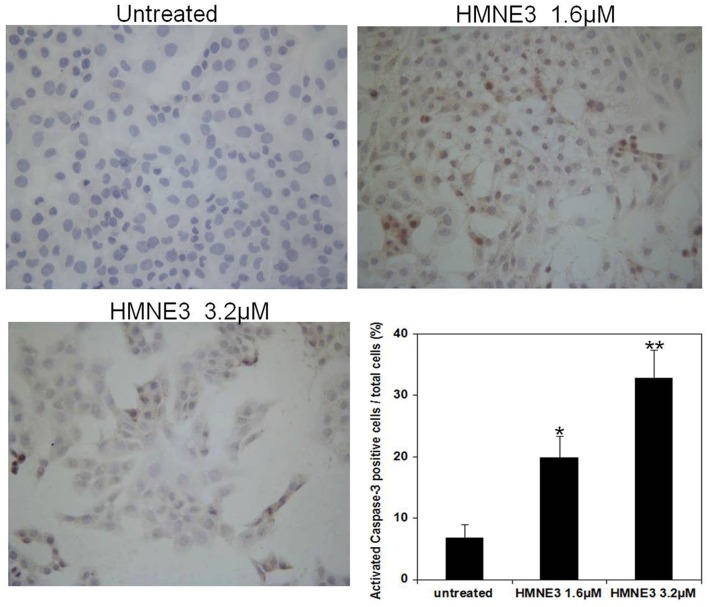
Immunocytochemical staining of activated Caspase-3 in Capan-1 cells following HMNE3 treatment. Approximately 2×10^4^ cells were plated on coverslips that were pre-placed in 12-well plates. All of the cells were treated with the indicated doses of HMNE3 for 48 h. The cells were fixed and incubated with 5% BSA for 30 min. The cells were incubated with a rabbit anti-human cleaved Caspase-3 antibody overnight at 4°C, washed with PBS, and incubated with a biotinylated horse anti-rabbit IgG antibody for 30 min. After rinsing with PBS for 5 min, streptavidin-horseradish peroxidase enzyme complex was diluted in PBS and incubated with the cells for 30 min. After washing with PBS, the staining was visualized by incubating the cells in DAB and counter-stained with a hematoxylin solution. The cells were imaged using a microscope. * P<0.05 compared with the control group; Student’s t test.

Cellular apoptosis was induced through intrinsic and/or extrinsic pathways due to persistent mitotic arrest or DNA damage. To explore the mechanisms involved in the cell growth inhibition of HMNE3, we detected the morphological changes in the cells upon HMNE3 treatment. As shown in Fig 4, we demonstrated that 3.2 μmol/L HMNE3 significantly increased the apoptosis of cultured Capan-1 cells. Moreover, increased TUNEL staining (Fig 5) was observed in the HMNE3-treated group. As one of the most important shear enzymes in apoptosis, Caspase-3 plays a critical role in the apoptotic cascade. Therefore, in our present study, immunocytochemical staining with an anti-cleaved Caspase-3 antibody was used to evaluate the levels of apoptosis. The expression of cleaved Caspase-3 was increased when the cells were treated with HMNE3 for 48 h (Fig 6). Similar results were also observed in Panc-1 cells (S1–S3 Figs). These results indicated that HMNE3 directly inhibited cancer cell growth by inducing apoptosis.

### Effect of HMNE3 on tyrosine kinase activity

**Fig 7:**
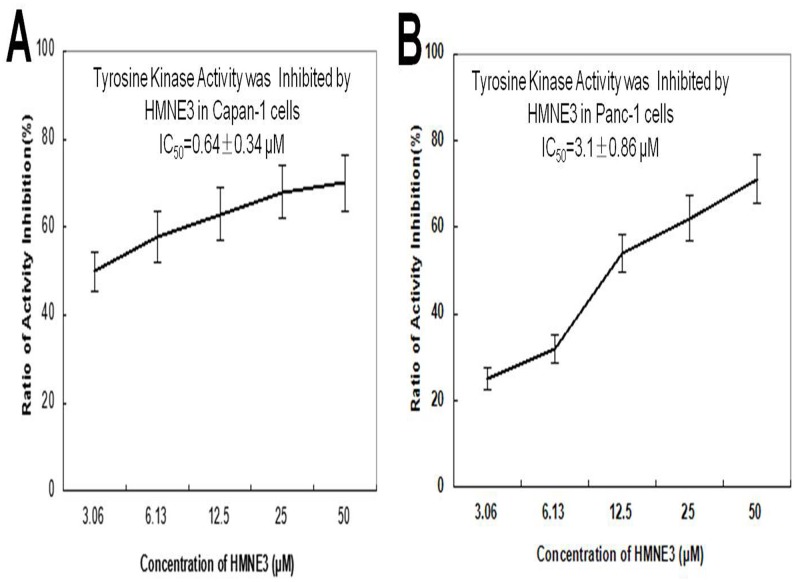
HMNE3 inhibited tyrosine kinase activity in the Capan-1 (A) and Panc-1 cells (B). Ninety-six-well plates were pre-coated with 20 μg/mL of a 4:1 poly (Glu,Tyr) solution as a substrate. An ATP solution was added to each well. Varying doses of HMNE3 were added to each test well. The kinase reaction was initiated by the addition of purified tyrosine kinase proteins diluted in 49 μL of kinase reaction buffer. After incubation for 1 h at 37°C, the plate was washed thrice with PBST. An anti-phosphotyrosine (PY99) antibody was then added. After a 30 min incubation at 37°C, the plate was washed thrice, and 100 μL of a horseradish peroxidase-conjugated goat anti-mouse IgG was added. The reaction was terminated by the addition of 50 μL of 2 mol/L H_2_SO_4_, and the plate was read at 490 nm using a multi-well spectrophotometer.

To determine the effect of HMNE3 on tyrosine kinase activity *in vitro*, Capan-1 or Panc-1 cells were treated with the indicated amounts of HMNE3 for the indicated times. Whole cell extracts were extracted and incubated with tyrosine kinase substrates, according to the manufacturer’s instructions. Fig 7 indicates that HMNE3 induced a dose-dependent decrease in the tyrosine kinase activity in Capan-1 cells. Similar results were observed in Panc-1 cells. These findings suggest that the cell cycle arrest induced by HMNE3 may be related to its inhibition of tyrosine kinase activity.

### Effects of HMNE3 on eukaryotic topoisomerase II activity

**Fig 8:**
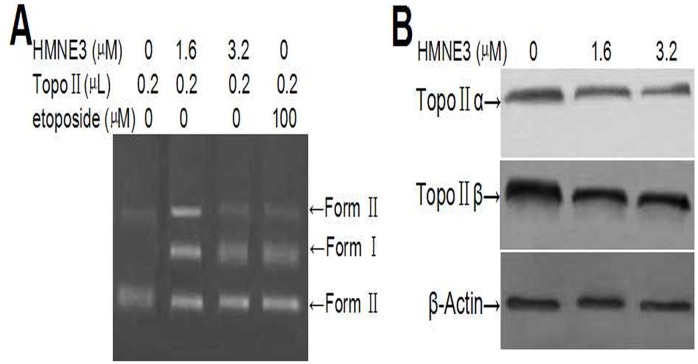
*In vitro* Topo II activity assay using agarose gel electrophoresis and western blotting. (A) Approximately 0.25 μg pBR322 DNA was incubated with 1 unit of human Top IIα and 1.6 μM HMNE3 for 30 min at 37°C in a total of 20 μL of reaction buffer. The reaction was then stopped with 2 μL of 10% SDS and 1 μL proteinase K. The samples were subjected to electrophoresis on a 0.8% agarose gel in 1× TAE at 5 V/cm for 1 h. Then, the gel was stained with 0.5 μg/mL of ethidium bromide (EB) for 30 min, destained with distilled water for 30 min, and photographed under a UV trans-illuminator. (B) The expression levels of Top IIα and Top IIβ were detected by western blotting.

The relative amounts of double-stranded DNA cleavage/religation were determined using an agarose gel electrophoresis method (Fig 8A). HMNE3 decreased the amount of supercoiled DNA (Form I) and increased the amount of nicked circular plasmid molecules (Form II) and linear molecules (Form III) in a dose-dependent manner. This result suggests that HMNE3 increased topoisomerase II-mediated DNA double-strand breaks and inhibited DNA religation.

Cancer cells can acquire resistance to topoisomerase II inhibitors by reducing their expression and/or activity of topoisomerase II. Therefore, we also detected the expression of topoisomerase II following HMNE3 treatment. Reduced levels of both Top IIα and Top IIβ were observed in Capan-1 cells treated with 1.6 μmol/L or 3.2 μmol/L HMNE3 for 24 h (Fig 8B).

**Fig 9:**
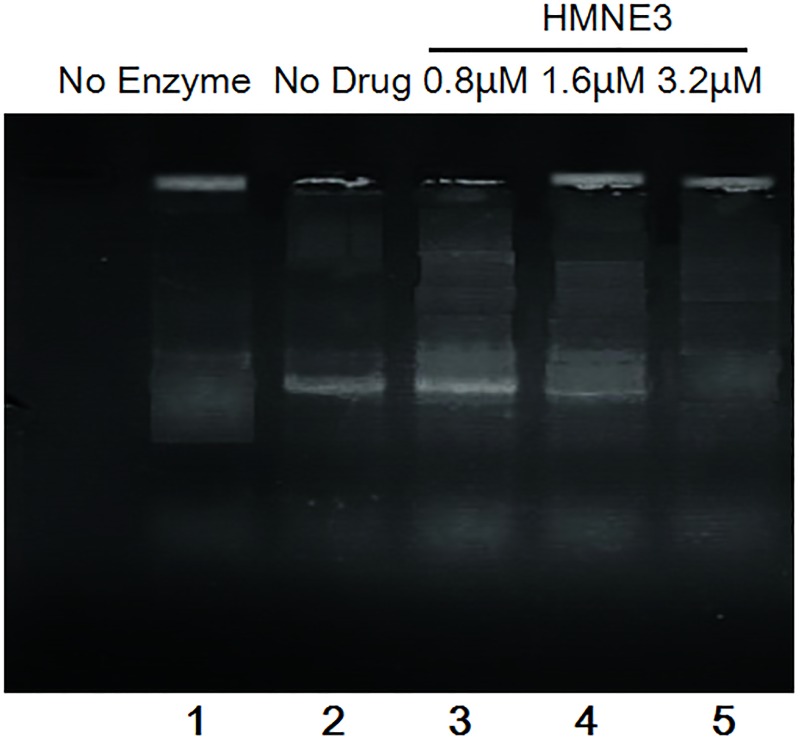
Inhibition of decatenation activity of topoisomerase II by HMNE3. All the cells were harvested on ice and nuclear extracts were separated from whole cellular proteins. Total 0.1 μg kDNA and 1unit of human topo IIa were mixed with the reaction buffer at 37°C for 15 min, the reaction was stopped by the addition of 10% SDS. Then, the DNA samples were subjected to electrophoresis in a 1% agarose gel containing 1 ug/ml ethidium bromide.

HMNE3 inhibited the decatenation of kinetoplast DNA by topoisomerase II (Fig 9, Lanes 4,5). Partial inhibition was evident at 1.6μM, with virtually complete inhibition at 3.2μM with HMNE3.

**Fig 10:**
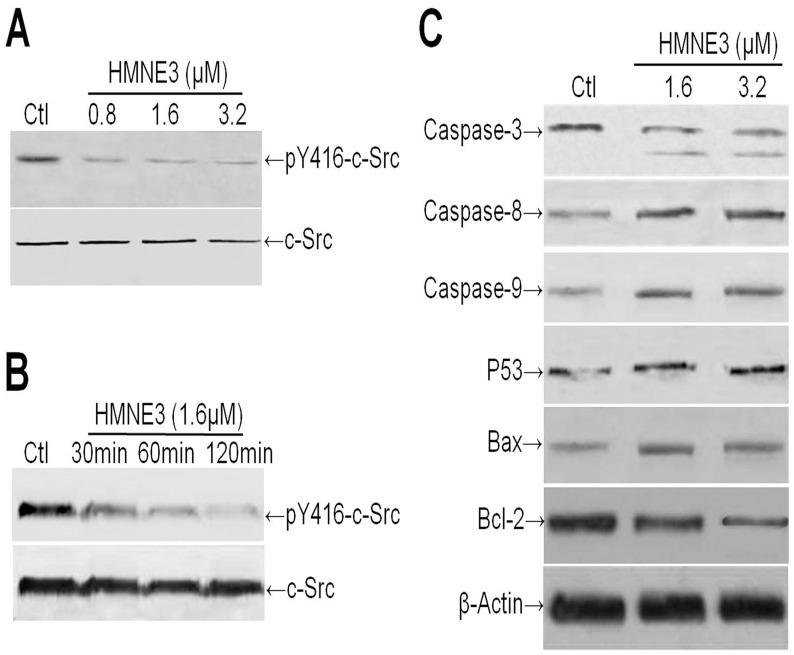
HMNE3 decreased the activity of c-Src and the cellular stress response. (A) The activity of c-Src was significantly inhibited by HMNE3 in a dose- and time-dependent manner (B). The expression levels of cell stress response-related proteins and proteins that regulate apoptosis were detected by western blotting (C).

To evaluate the effect of HMNE3 on the activity of c-Src, Capan-1 cells were treated with varying concentrations of HMNE3 for 15, 30 or 60 min. The c-Src activity was determined using a phospho-specific antibody against the c-Src autophosphorylation site (pY416). The result demonstrated that HMNE3 exposure reduced the level of activated c-Src (pY416) in a time- and dose-dependent manner (Fig 10A and 10B).

To study the mechanism of HMNE3-induced apoptosis, we next assessed Caspase activation by western blot analysis. Treatment with HMNE3 increased the levels of cleaved Caspase-9 and -3 in the Capan-1 cells, indicating that these caspases were involved in apoptosis induction (Fig 10C). The pathway leading to the induction of apoptosis also involves p53, Bax (proapoptotic) and the Bcl-2 family (antiapoptotic). We next assayed the expression levels of p53, Bax and Bcl-2 proteins in the presence of HMNE3. Fig 10C reveals up-regulation of p53 and Bax expression in the Capan-1 cells after 48 h of HMNE3 treatment. However, Bcl-2 expression significantly decreased.

## Discussion

Clinical investigations revealed that chemotherapy has little effect on human pancreatic cancer (HPAC) due to side effects and multidrug resistance [16,17]. Therefore, it is very urgent to develop new drugs to treat HPAC.

Previous studies demonstrated that fluoroquinolone compounds inhibit carcinoma cell proliferation by inducing apoptosis *in vitro* [18,19]. A number of chemically modified antibacterial fluoroquinolone derivatives have been developed. These chemicals exhibit enhanced activity against mammalian topoisomerase II and inhibit DNA replication [20, 21]. These fluoroquinolone derivatives share a similar mechanism of action with several clinically relevant antitumor agents [22]. In our current study, we first synthesized dual topoisomerase II and tyrosine kinase inhibitors based on the structural features of fluoroquinolone and sunitinib. Our study may help develop new multi-target drugs against human pancreatic cancer therapy.

Of these derivatives, some drugs, such as HMNE3 have a potential role in inhibiting cancer cell proliferation *in vitro*. HMNE3 treatment inhibited cell survival and the proliferation of pancreatic cancer cells by inducing apoptosis *in vitro*. Tyrosine kinases are involved in cancer cell growth, colony formation and angiogenesis [23, 24]. However, the signaling pathways triggered by these receptors can be blocked by small-molecule inhibitors of receptor-associated tyrosine kinases. Therefore, the HMNE3-mediated inhibition of the proliferation of Capan-1 and Panc-1 cells may occur through the regulation of tyrosine kinase activity. The HMNE3 treatment resulted in reduced c-Src phosphorylation as well as impaired cellular function in the pancreatic cancer cells *in vitro*. Furthermore, HMNE3 inhibited the ability of Top II to relax supercoiled pBR322 DNA. Top II inhibition may be the direct factor that causes cellular apoptosis. HMNE3 treatment induced apoptosis in Capan-1 and Panc-1 cells. Cellular apoptosis was initiated by the cascading activation of caspases. HMNE3 up-regulated the expression of Caspase-3/8/9 to induce apoptosis.

Thus, these results indicated that HMNE3 is a potential multi-target inhibitor of tyrosine kinases and Top II. Most importantly, the information obtained from our study could be useful for the development of novel therapeutic drugs for the treatment of pancreatic cancer.

## Supporting Information

S1 Fig Nuclear staining of Panc-1 cells following a 48 h treatment with HMNE3. (TIF)

S2 Fig TUNEL staining of Panc-1 cells following a 48 h treatment with HMNE3. (TIF)

S3 Fig Immunocytochemical staining of activated Caspase-3 in Panc-1 cells following HMNE3 treatment. *, P<0.05 compared with the control group; Student’s t test.
(TIF)

S4 Fig Effect of Sunitinib on the growth of HPAC and Capanc-1 cells by MTT assay. (TIF)

S5 Fig Effect of Sunitinib on the Tyrosine Kinase Activity of HPAC and Capanc-1 cells. (TIF)
