# Expression of Concern: Dual Inhibition of Topoisomerase II and Tyrosine Kinases by the Novel Bis-Fluoroquinolone Chalcone-Like Derivative HMNE3 in Human Pancreatic Cancer Cells

**DOI:** 10.1371/journal.pone.0294622

**Published:** 2023-11-15

**Authors:** The PLOS ONE Editors

Following the publication of this article [1], concerns were raised regarding the results in multiple figures. Specifically,

- The pY416-c-Src panel in Fig 10B of this article [1] appears similar to an image used to represent HIF-1α in SKOV3 cells in Fig 3B of a later article with no shared authors [2].
- The HMNE3 0.8 μM panels in Fig 5 and S2B Fig appear to partially overlap despite representing different cell lines.
- In 9, there appear to be horizontal and vertical discontinuities in multiple lanes.

PLOS did not receive a response from the authors, and in the absence of clarifications or original underlying data, the issues cannot be resolved.

In addition, the primary data underlying results in this article were not included with the published article although the Data Availability statement for this article stated, “All relevant data are within the paper and its Supporting Information files.”

The *PLOS ONE* Editors issue this Expression of Concern to inform readers about these issues which raise concerns regarding the reliability of the affected data.
