# Supplementary figures and images for: Dual Inhibition of Topoisomerase II and Tyrosine Kinases by the Novel Bis-Fluoroquinolone Chalcone-Like Derivative HMNE3 in Human Pancreatic Cancer Cells
Source: PLoS One. 2016 Oct 19;11(10):e0162821. doi: 10.1371/journal.pone.0162821 (PMC5070812; doi:10.1371/journal.pone.0162821)

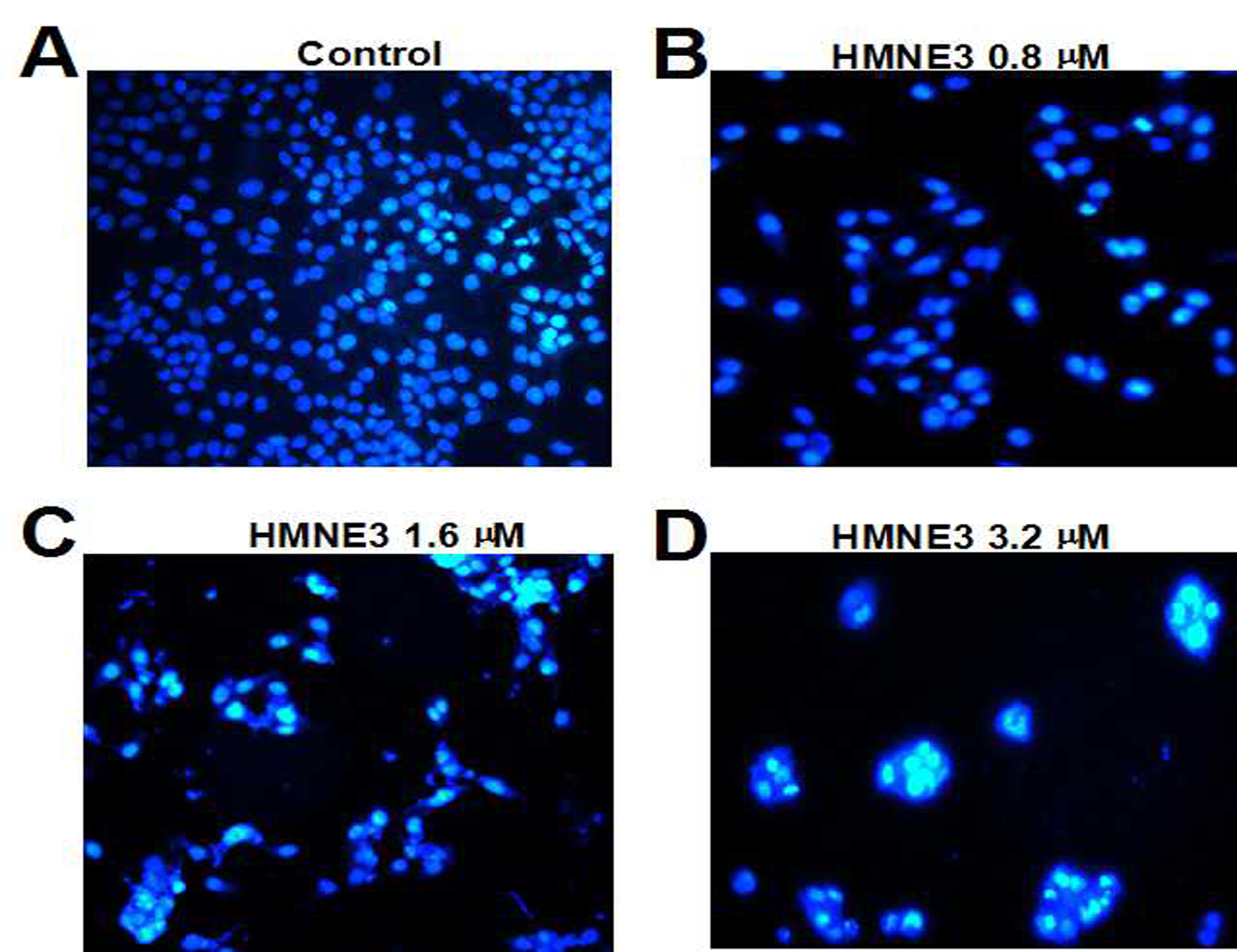

Supplement: S1 Fig — (TIF) [file pone.0162821.s001.tif]

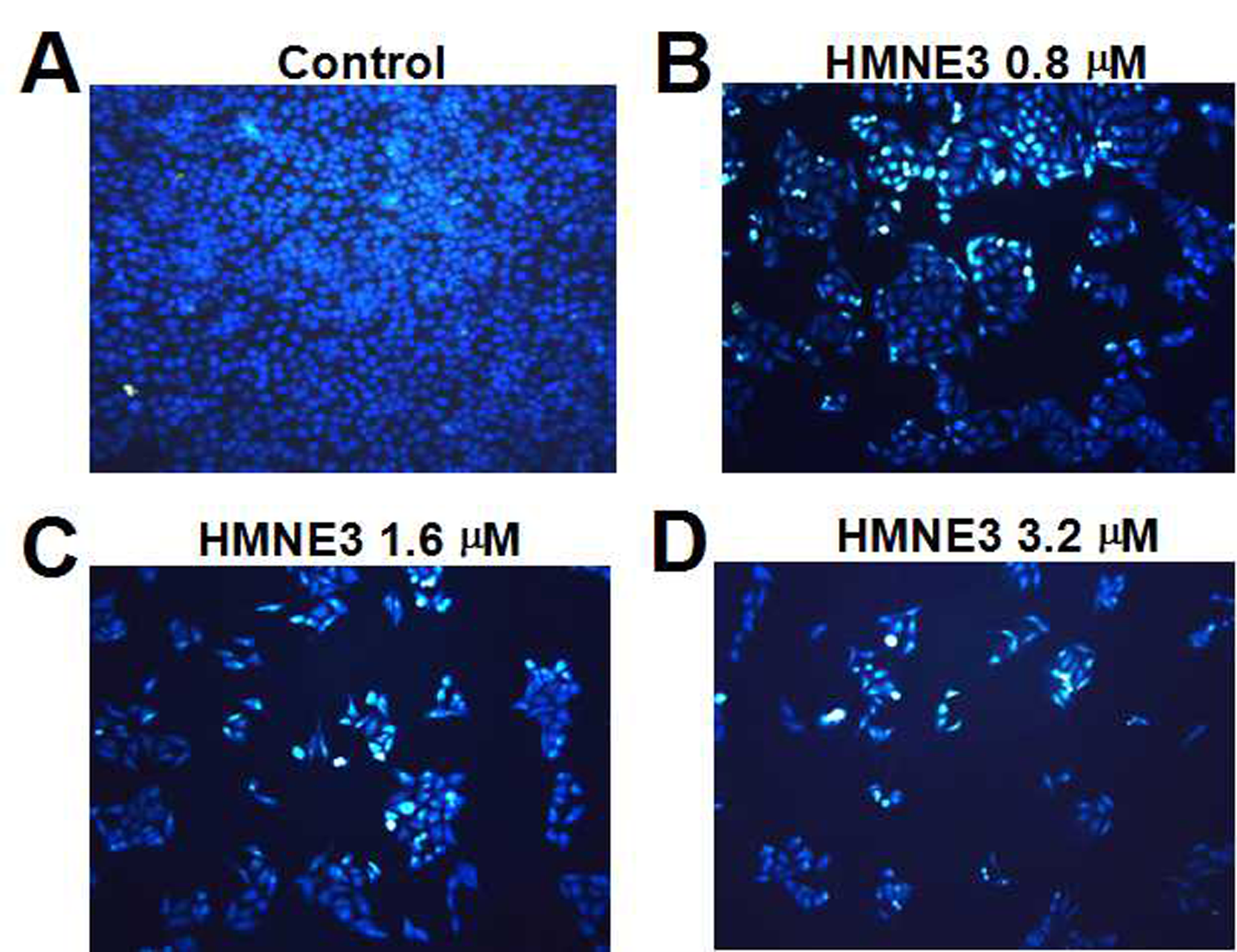

Supplement: S2 Fig — (TIF) [file pone.0162821.s002.tif]

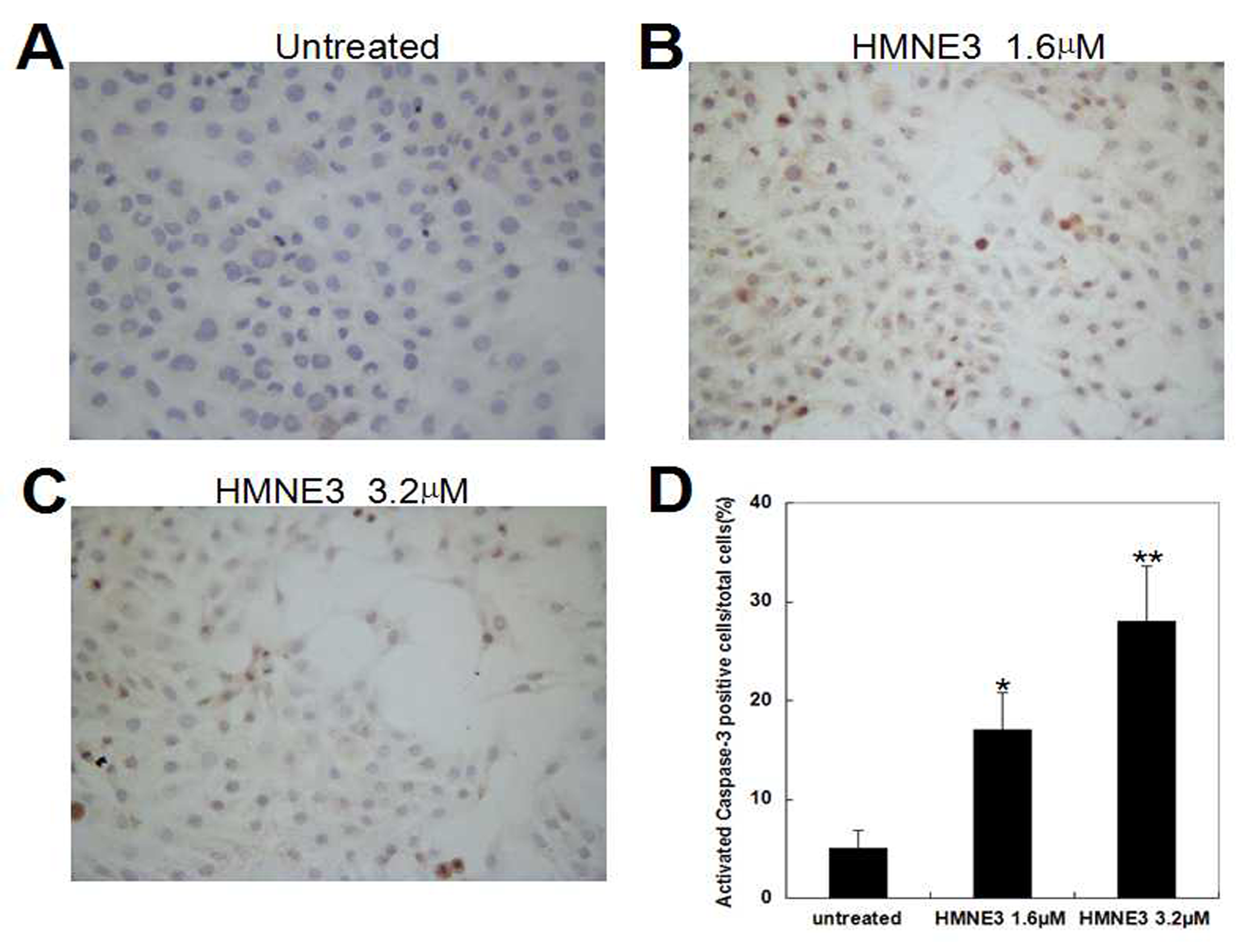

Supplement: S3 Fig — *, P<0.05 compared with the control group; Student’s t test. (TIF) [file pone.0162821.s003.tif]

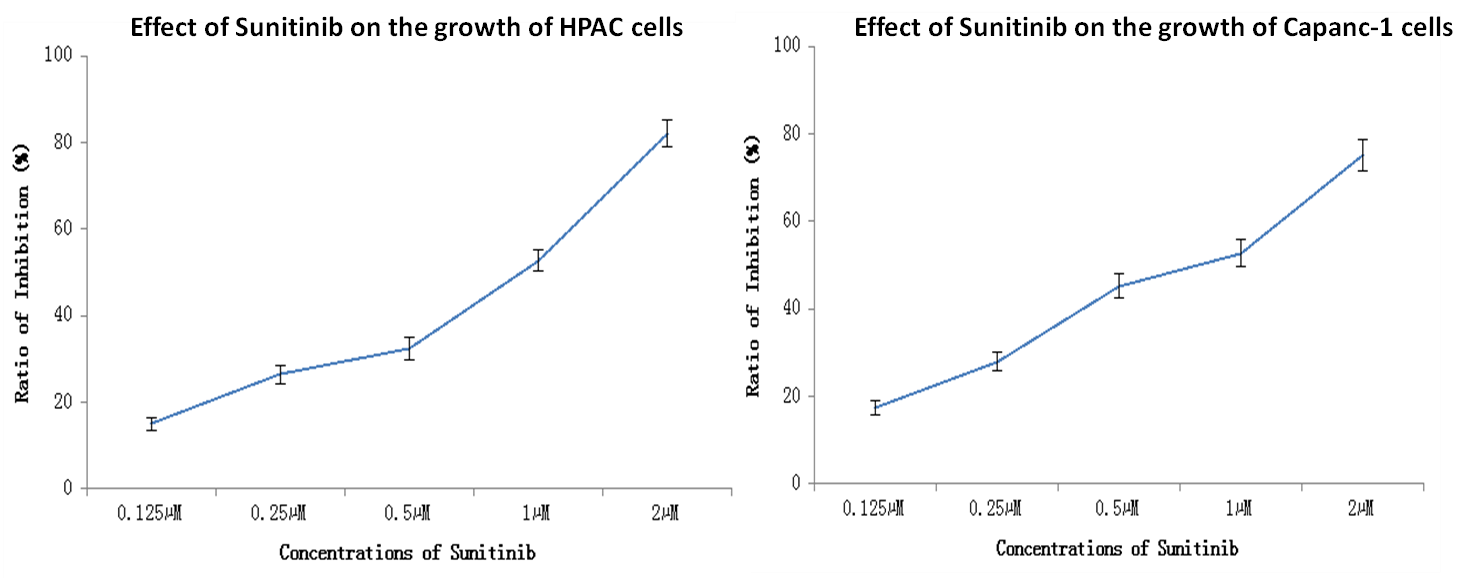

Supplement: S4 Fig — (TIF) [file pone.0162821.s004.tif]

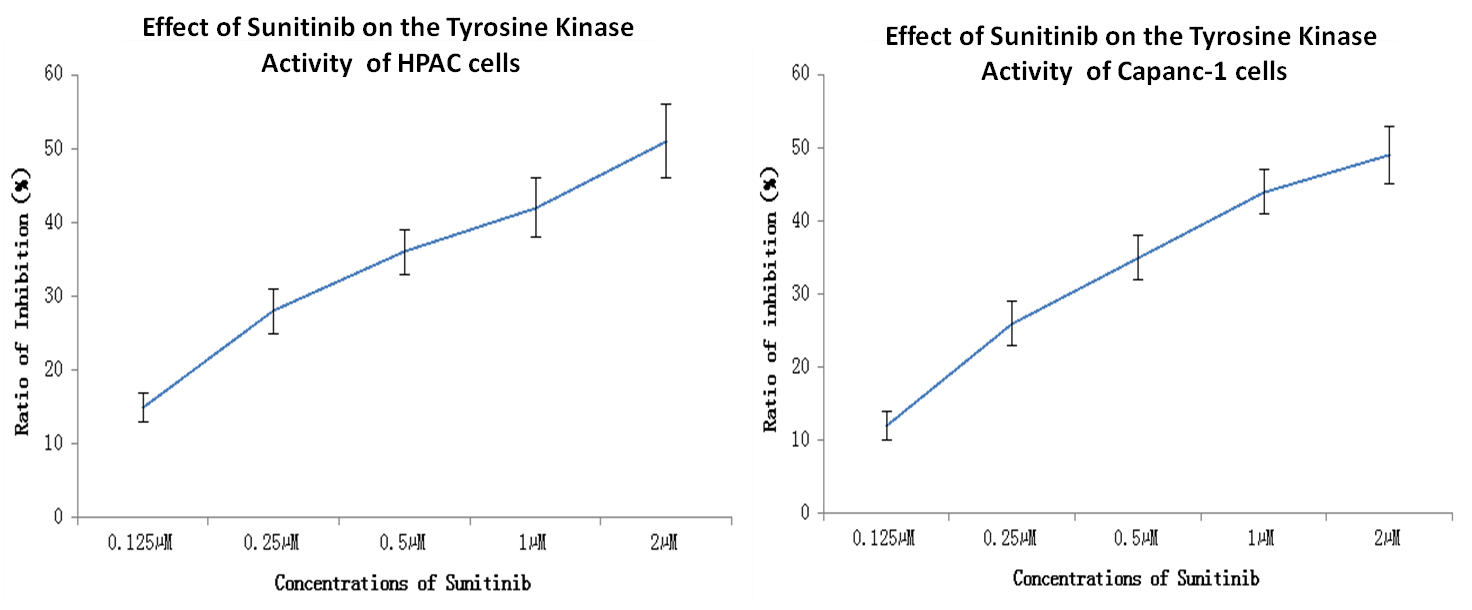

Supplement: S5 Fig — (TIF) [file pone.0162821.s005.tif]
